# Effectiveness of culturally appropriate antenatal education packages to improve birth preparedness and complication readiness in low- and middle-income countries: a systematic review

**DOI:** 10.1186/s12884-026-09265-0

**Published:** 2026-05-18

**Authors:** Zeenat Moromoke Abdulwahab, Chika Chioma Harriet Odira, Grace Daniel, Cosmos Enyindah

**Affiliations:** 1 Department of Midwifery, African Centre of Excellence for Public Health and Toxicological Research (ACEPUTOR), University of Port Harcourt, Port Harcourt, Rivers State Nigeria; 2 Department of Nursing Science, Nnamdi Azikiwe University, Awka, Anambra State Nigeria; 3 Department of Maternal and Child Health, University of Jos, Jos, Plateau State Nigeria; 4 Department of Obstetrics and Gynecology, University of Port Harcourt, Port Harcourt, Rivers State Nigeria

**Keywords:** Birth preparedness, Complication readiness, Antenatal education, Low- and middle-income countries, Maternal health, Systematic review

## Abstract

**Background:**

Birth preparedness and complication readiness (BPCR) remain essential components of maternal survival strategies in low- and middle-income countries (LMICs). Suboptimal antenatal education contributes significantly to delayed recognition of obstetric danger signs and late care-seeking. Delays in recognising obstetric danger signs and seeking care contribute to high maternal mortality in LMICs. Culturally tailored antenatal education packages have been increasingly used to improve BPCR; however, evidence across LMICs has not been systematically synthesised.

**Objective:**

To synthesise evidence on the effectiveness of culturally appropriate antenatal education interventions on BPCR outcomes among pregnant women in LMICs.

**Methods:**

This systematic review adhered to PRISMA 2020 and JBI guidelines. We searched PubMed, Scopus, Web of Science, CINAHL, AJOL, and Google Scholar (January 2015–July 2025) for English-language studies on antenatal education interventions reporting BPCR outcomes in LMICs. Two reviewers screened titles/abstracts, assessed full texts, appraised quality (JBI checklists), and extracted data for narrative synthesis due to heterogeneity.

**Results:**

Eleven studies (*n* = 5,802; quasi-experimental *n* = 4, cross-sectional *n* = 4, cohort *n* = 2, community-based *n* = 1) from Ethiopia (3), Nigeria (3), Tanzania (2), Cameroon (1), Kenya (1), Bangladesh (1) showed consistent improvements: danger sign knowledge up to 85% (mobile SMS, Tanzania), BPCR practices 25–95% (e.g., goal-oriented education, Nigeria: 65.5% to 95.3%), institutional delivery 53.5% to 93.5%. Predictors include early ANC, partner involvement and local languages. Quality: low-moderate risk of bias.

**Conclusions:**

Culturally matched antenatal classes help pregnant women in low-income countries better prepare for childbirth and emergencies, improving knowledge by up to 85% and care-seeking behaviour by 25–95%. This review of 11 studies supports the use of local languages, family involvement, and phone messages in routine prenatal care to save lives. More high-quality trials are needed.

**Supplementary Information:**

The online version contains supplementary material available at 10.1186/s12884-026-09265-0.

## Introduction

Maternal mortality ratio in LMICs remains 211 per 100,000 live births [1], driven by three delays: deciding to seek care, reaching facilities, and receiving treatment. BPCR, which includes planning skilled attendants, transport, funds, and danger sign recognition, mitigates these [2].

Birth Preparedness and Complication Readiness (BPCR) is widely recognised as a core safe-motherhood strategy that encourages women, families, and communities to plan proactively for skilled birth attendance, emergency transport, and financial readiness for obstetric complications [3–6].

Antenatal care (ANC) provides a key platform for delivering such information; however, in many LMICs, education during ANC remains largely generic, didactic, and poorly aligned with women’s sociocultural realities, contributing to persistently low BPCR indicators despite relatively high ANC attendance [6, 7]. In response, several LMICs have implemented culturally tailored antenatal and maternal education packages ranging from structured group sessions and community health worker-led outreach to mobile/SMS interventions and family-inclusive models designed to reflect local norms, languages, belief systems, and social structures, yet understanding the effectiveness of these approaches remains essential for strengthening maternal and newborn health interventions and guiding scale-up [7, 8, 9].

This systematic review synthesises current evidence (2015–2025) on culturally appropriate antenatal education interventions aimed at improving BPCR in LMIC settings, using the PICO framework [10]. PICO stands for Population, Intervention, Comparator, Outcome. P =pregnant women in LMICs; I =tailored education; C =routine; O =BPCR knowledge, practice, and utilisation.

## Methods

### Protocol and reporting

The protocol was prospectively registered (PROSPERO CRD4201130990). This review followed the Joanna Briggs Institute (JBI) evidence synthesis approach and PRISMA 2020 reporting guidelines [11, 12].This review was conducted and reported in accordance with the PRISMA 2020 guidelines and outlined the objectives, eligibility criteria, search strategy, and planned analyses.

(Appendix A)

### Eligibility criteria

Population: Pregnant women or recently delivered women residing in low- and middle-income countries (LMICs).

Intervention: Culturally adapted antenatal education interventions, delivered in facility-based, community-based, or mixed settings, including structured classes, mobile/SMS programmes, or family-inclusive approaches.

Comparator: Routine or standard antenatal education/care.

Outcomes: Birth preparedness and complication readiness (BPCR), including knowledge of obstetric danger signs, planning for skilled birth attendance, emergency transport, financial preparedness, and facility delivery.

Study Designs: Quasi-experimental, cross-sectional, cohort, and mixed-methods studies published in English.

Period: 2015–2025. The starting point of 2015 was chosen to capture evidence after the WHO 2015 antenatal care recommendations and to reflect contemporary interventions in LMICs.

Exclusion: Studies conducted outside LMICs, case reports, commentaries, or studies lacking BPCR outcomes were excluded. Studies that focused solely on maternal health education without measurable BPCR outcomes were also excluded.

### Search strategy

A comprehensive search was conducted in PubMed, Scopus, Web of Science, CINAHL, AJOL, and Google Scholar. Boolean combinations included: “antenatal education” AND “birth preparedness” AND “complication readiness” AND “LMICs” and synonyms. Search dates ranged from January 2015 to July 2025. Reference lists of relevant studies were hand-searched to identify additional eligible articles (Appendix B). No additional filters were applied beyond publication date and language restrictions.

### Screening and selection process

After duplicate removal, two independent reviewers (ZMA and CCHO) screened titles and abstracts, followed by full-text review. Discrepancies were resolved through discussion and consultation with a third reviewer. The selection process excluded studies with ineligible designs (e.g., protocols, editorials, reviews) or without measurable BPCR outcomes. Exclusion reasons were pre-specified in the protocol.

### Data extraction

Data were extracted using a standardised JBI-based form, capturing study characteristics (author, year, country, design, sample size), intervention features (type, duration, delivery method, degree of cultural adaptation), BPCR outcomes measured, and key findings (appendix C). Data extraction was conducted independently by two reviewers (ZMA and CCHO) to ensure accuracy, with consensus reached through discussion.

### Quality appraisal (Risk of bias assessment)

Study quality was assessed using the JBI critical appraisal checklists, which consider sample selection, measurement validity, statistical analysis, and reporting transparency. Low to moderate risk of bias was observed across studies.

All included studies met the pre-specified quality threshold of ≥ 70% positive appraisal criteria using the JBI checklists. Common methodological limitations included the absence of randomisation (in quasi-experimental designs), reliance on self-reported BPCR measures, and limited follow-up duration. However, outcome reporting and statistical analyses were generally adequate. Overall, the body of evidence was assessed as low to moderate risk of bias. A detailed appraisal summary is presented in Appendix D.

### Definition and measurement of effectiveness

In this review, “effectiveness” was defined as measurable improvement in one or more Birth Preparedness and Complication Readiness (BPCR) outcomes following exposure to a culturally adapted antenatal education intervention. These outcomes included: (1) increased knowledge of obstetric danger signs, (2) improved BPCR practice indicators (e.g., financial planning, transport arrangement, identification of skilled birth attendants), and (3) increased utilisation of institutional delivery services [7]. Effectiveness was assessed using percentage change between baseline and post-intervention assessments, odds ratios, adjusted associations, or statistically significant differences, as reported in the primary studies.

Outcome measurement was not standardised across studies. Some studies used composite BPCR indices, whereas others assessed individual preparedness components or knowledge scores. Given these variations, effectiveness was interpreted comparatively rather than statistically pooled [13].

### Data synthesis

Given substantial heterogeneity in study design, intervention structure, outcome definitions, and reporting metrics, quantitative pooling through meta-analysis was not feasible. Included studies comprised quasi-experimental, cross-sectional, cohort, and community-based intervention designs, with variable definitions of BPCR and inconsistent measurement approaches. This methodological diversity precluded statistical aggregation.

A structured narrative synthesis approach was therefore undertaken. First, studies were grouped by intervention type (facility-based, community-based, digital/mobile, and family-inclusive). Second, study characteristics and outcomes were tabulated to facilitate structured comparison. Third, findings were organised into predefined outcome domains: (1) knowledge of obstetric danger signs, (2) BPCR practices, (3) institutional delivery and skilled birth attendance, and (4) predictors and contextual modifiers. Finally, cross-study comparisons were conducted to identify patterns of consistency and divergence.

Data extraction was performed independently by two reviewers, and synthesis decisions were reached through consensus discussions.

Narrative synthesis was selected over thematic synthesis because the included studies primarily reported quantitative outcomes rather than qualitative themes [14]. No meta-analysis was conducted because of methodological variability [15]. The synthesis was guided by the social-ecological framework, emphasising individual, household, and community-level influences on BPCR outcomes. GRADE assessment indicated moderate certainty for primary outcomes.

## Results

### Study Selection

The database search yielded a total of 1,268 records from PubMed, Scopus, Web of Science, CINAHL, AJOL, and Google Scholar. After the removal of 304 duplicates, 964 records were screened by title and abstract. Of these, 892 records were excluded for irrelevance to antenatal education or BPCR outcomes. Full-text assessment was conducted for 72 articles, of which 61 were excluded due to non-LMIC settings, lack of BPCR-related outcomes, or inappropriate study designs. A total of 11 studies met the inclusion criteria and were included in the final synthesis. The study selection process is summarised in the PRISMA 2020 flow diagram (Fig. 1). See Appendix E.

### Characteristics of included studies

The 11 included studies were conducted across six low- and middle-income countries: Ethiopia (*n* = 3), Nigeria (*n* = 3), Tanzania (*n* = 2), Cameroon (*n* = 1), Kenya (*n* = 1), and Bangladesh (*n* = 1). Study designs comprised cross-sectional surveys (*n* = 4), quasi-experimental studies (*n* = 4), cohort studies (*n* = 2**)**, and community-based intervention studies (*n* = 1). Sample sizes ranged from 120 to over 1,200 participants, and all studies focused on pregnant women attending antenatal care or women who had recently delivered. Interventions varied from goal-oriented prenatal education, mobile/SMS-based messaging, family-inclusive group sessions, and routine ANC counselling. Cultural adaptations included local languages, engagement of husbands or family members, and use of community-relevant examples [3–9, 16–19]. A summary of the included studies, including setting, design, intervention type, and key outcomes, is presented in Table 1 below.

**Table 1:** Characteristics of Studies Included in the Systematic Review

Author (Year)	Country	Study Design	Study Setting	Sample Size	Intervention	Key BPCR Outcomes
Masoi & Kibusi (2019)	Tanzania	c	Health facilities	450	Mobile phone–based antenatal education using culturally adapted SMS messages	Knowledge of danger signs + 85%, BPCR + 90%
Akinwaare & Oluwatosin (2023)	Nigeria	Quasi-experimental	Semi-urban ANC clinics	372	Goal-Oriented Prenatal Education (GOPE) delivered in group sessions	BPCR 65.5→95.3%; institutional delivery 53.5→93.5%
Letose et al. (2020)	Ethiopia	Cross-sectional	Community-based	670	Routine antenatal counselling	BPCR practices + 25.8%
Moinuddin et al. (2017)	Bangladesh	Community-based intervention	Community	1,200	Community health education with family involvement	Facility delivery OR 2.4; BPCR
Shimpuku et al. (2019)	Tanzania	Cohort	Health facilities	300	Family-oriented antenatal education programmeme	Improved Birth preparedness
Debelie et al. (2021)	Ethiopia	Cross-sectional	Community-based	590	ANC-based maternal health education	Better BPCR knowledge and practice
Girma et al. (2022)	Ethiopia	Cross-sectional	Health facilities	760	Routine ANC counselling	Early ANC and BPCR practice
Ijang et al. (2021)	Cameroon	Cross-sectional	Health facilities	450	Facility-based antenatal education	Higher BPCR
Ihomba et al. (2020)	Kenya	Cohort	Health facilities	240	Antenatal education during routine ANC	BPCR; reduced maternal adverse outcomes
Obionu et al. (2022)	Nigeria	Cross-sectional	Health facilities	500	Antenatal health education sessions	BPCR
Imaralu et al. (2020)	Nigeria	Cohort	Health facilities	320	Antenatal education and counselling	BPCR: better perinatal outcomes

Table 1 summarizes 11 studies from LMICs evaluating antenatal education interventions, primarily culturally adapted ones, that enhance birth preparedness and complication readiness (BPCR) among pregnant women. Each study links specific interventions to measurable BPCR improvements, such as increased knowledge of danger signs, better practices (e.g., saving money/transport, identifying delivery sites), and higher institutional delivery rates.

These interventions typically boost BPCR via tailored education, family involvement, or mobile/community delivery, with quasi-experimental designs showing the strongest gains.

Quasi-experimental study by Masoi & Kibusi (2019) in Tanzania, shows that mobile phone SMS with culturally adapted messages raised danger sign knowledge by 85% and overall BPCR by 90% in 450 facility-based women, leveraging accessible tech for repeated reinforcement [16]. Quasi-experimental study conducted in Nigeria by Akinwaare & Oluwatosin (2023) using Group-based Goal-Oriented Prenatal Education (GOPE) in semi-urban clinics improved BPCR scores from 65.5% to 95.3% (372 women) and institutional deliveries from 53.5% to 93.5%, emphasizing goal-setting for planning [17]. In a Cross-sectional study in Ethiopia, Letose et al. (2020) noted that routine community counseling increased BPCR practices by 25.8% among 670 women, highlighting the role of basic counseling in awareness [18]. Moinuddin et al. (2017) observed, during a community-based intervention in Bangladesh, that family-based community education yielded an odds ratio of 2.4 for facility delivery and general BPCR gains among 1,200 participants via social support networks [19]. Shimpuku et al. (2019) in Tanzania demonstrated that a family-oriented antenatal program enhanced birth preparedness among 300 facility attendees by focusing on household decision-making [20]. Debelie et al. (2021) in Ethiopia found that ANC maternal health education improved BPCR knowledge and practice among 590 community women through targeted health talks [21]. Similarly, Girma et al. (2022) in Ethiopia showed that routine ANC counseling promoted early visits and BPCR practice in 760 facility-based women by linking attendance to readiness [22]. In Cameroon, Ijang et al. (2021) reported that facility-based education led to higher overall BPCR in 450 women via structured sessions [23]. Ihomba et al. (2020) in Kenya demonstrated that routine ANC education improved BPCR and reduced adverse outcomes in 240 facility women by integrating readiness into visits [24]. Obionu et al. (2022) in Nigeria observed that health education sessions directly advanced BPCR in 500 facility women by focusing on key elements [25]. Finally, Imaralu et al. (2020) in Nigeria showed that education and counseling enhanced BPCR and perinatal outcomes in 320 facility women, emphasizing complication planning [26].

### Effects of antenatal education on knowledge of obstetric danger signs

Across the included studies, three studies show that antenatal education interventions improve women’s knowledge of obstetric danger signs. A mobile phone-based education intervention in Tanzania reported an 85% increase in knowledge following repeated messaging [16]. In Nigeria, structured goal-oriented prenatal education resulted in an increase in correct identification of danger signs from 65.5% at baseline to 91.8% post-intervention [17]. Similar improvements were reported in Ethiopia, where community-based education interventions improved knowledge scores by more than 30% points in some settings.

### Effects on birth preparedness and complication readiness (BPCR) practices

All included studies reported improvements in BPCR practices following antenatal education interventions. Improvements ranged from 25.8% in a community-based study in Ethiopia [18] to 95.3% following a goal-oriented prenatal education programme in Nigeria [17]. Key BPCR components that improved included identifying skilled birth attendants, arranging emergency transport, ensuring financial preparedness, and identifying a health facility for delivery. Studies employing structured, culturally adapted content demonstrated larger effect sizes than those observed with routine or unstructured antenatal education.

### Institutional delivery and skilled birth attendance

Seven studies assessed the relationship between antenatal education and utilisation of skilled birth attendants or facility-based delivery. A community-based intervention in Bangladesh reported that women exposed to antenatal education were 2.4 times more likely to deliver in a health facility compared to unexposed women [19]. In Nigeria and Ethiopia, facility delivery rates increased from approximately 53.5% to over 90% following structured antenatal education interventions. These findings suggest that antenatal education contributes not only to knowledge gains but also to tangible changes in care-seeking behaviour.

### Predictors and moderators of BPCR outcomes

Early initiation of antenatal care was positively associated with improved BPCR practices in three studies [22, 24, 26]. Partner involvement was reported as a significant predictor in the Bangladesh community intervention [19] and in the Tanzanian family-oriented programme [20]. Notably, studies with family or partner engagement reported a markedly higher BPCR uptake: for example, in the Bangladesh intervention, women whose partners participated had 74% BPCR uptake compared to 41% among those with no partner involvement [19]. Similarly, the Tanzanian programme saw BPCR practice rates of 81% with family engagement versus 50% without [20]. These comparative figures highlight the substantial influence of household dynamics, making family participation one of the strongest moderators of intervention effectiveness. Exposure to structured goal-oriented education demonstrated stronger effects than routine counselling in Nigeria [17].

These factors appear to function as effect modifiers, enhancing the magnitude of BPCR improvements when integrated into the intervention design. Consistency across multiple LMIC settings suggests that early ANC engagement and family participation are robust contextual facilitators.

### Cultural and contextual features of effective interventions

Cultural adaptation was operationalised through several mechanisms across studies. These included delivery in local languages (e.g., Swahili SMS in Tanzania; Yoruba-based sessions in Nigeria), incorporation of culturally relevant examples and metaphors, inclusion of male partners or household decision-makers, and community-based outreach in settings where traditional birth norms influence care-seeking [13].

Family-inclusive sessions addressed patriarchal decision-making structures by engaging husbands in financial and transport planning [20]. Mobile interventions were tailored linguistically and contextually to reflect locally recognised danger signs [16]. These adaptations increased acceptability and relevance, thereby facilitating behavioural uptake beyond knowledge acquisition alone [17, 20].

## Discussion

This systematic review synthesises evidence on culturally appropriate antenatal education interventions aimed at improving birth preparedness and complication readiness (BPCR) among pregnant women in low- and middle-income countries (LMICs). A total of 5,802 participants were included across 11 studies. Interventions consistently improved knowledge of obstetric danger signs, BPCR practices, and utilisation of skilled birth attendants. Knowledge gains ranged from 30% to 85% [13–15], while improvements in BPCR practices ranged from 25.8% to 95.3% [17, 18]. Institutional delivery rates increased from 53.5% to over 90% in structured, culturally adapted programmes [17, 18, and 19].

### Interpretation of findings

Interventions demonstrated effectiveness when tailored to local sociocultural contexts. Cultural adaptation features included the use of local languages, context-specific examples, engagement of husbands or other family members, and delivery formats compatible with community norms (e.g., group sessions, home visits, mobile messaging) [16–20]. Interventions incorporating family involvement consistently yielded stronger and more sustained effects on BPCR, reflecting the influence of household decision-making dynamics in LMIC settings [17, 19].

Key Predictors of improved BPCR outcomes were early ANC initiation, ≥ 4 ANC visits, urban residence, and exposure to digital or mobile interventions [16–18]. These factors suggest that both intervention design and accessibility significantly influence effectiveness. The evidence indicates that structured and culturally relevant education packages outperform routine or unstructured ANC counselling, highlighting the need for contextually informed antenatal programmes [17, 18].

### Comparison with previous literature

Our findings align with the Johns Hopkins BPCR framework, emphasising planning for skilled birth attendance, emergency transport, and financial readiness [3]. This review extends prior work by including multiple LMICs and synthesising 11 studies across Africa and South Asia (Nigeria, Ethiopia, Tanzania, Cameroon, Kenya, and Bangladesh) [16–19]. Notably, the integration of mobile health interventions, such as SMS reminders and digital messages, is a recent innovation endorsed by the WHO 2025 guidelines to reach low-literacy populations [1, 16]. Previous systematic reviews largely focused on high-income settings or single-country interventions, making this review uniquely relevant for LMIC policy and practice [8, 13].

### Strengths and limitations

Strengths include: comprehensive database searches (PubMed, Scopus, Web of Science, CINAHL, AJOL, and Google Scholar), adherence to PRISMA 2020 and JBI guidance, dual independent screening and data extraction, and narrative synthesis that accounted for methodological heterogeneity [1, 2, and 11]. The inclusion of studies with diverse LMIC contexts enhances the generalizability of findings.

Limitations include reliance on self-reported outcomes, short follow-up durations (< 6 months), underrepresentation of Francophone West Africa, and absence of cost-effectiveness data. Additionally, quasi-experimental designs dominated, with few randomised trials, which may introduce selection bias [16–18]. Despite these limitations, the consistency of findings across multiple countries supports the robustness of the conclusions.

### Implications for policy, practice, and research

Practice: Culturally tailored antenatal education, including family involvement and mobile/digital messaging, should be integrated into routine ANC in LMICs. Programmes like goal-oriented prenatal education (GOPE) and family-inclusive community outreach are recommended for scale-up [16, 19].

Policy: Ministries of health in LMICs should fund context-specific antenatal education packages, monitor BPCR indicators through surveys (e.g., DHS), and integrate digital health interventions into national ANC programmes [27].

Research: Future studies should focus on high-quality cluster-RCTs, long-term follow-up to assess sustained BPCR outcomes, cost-effectiveness analysis, and equity evaluations in rural and indigenous populations. Investigating the additive effect of digital interventions combined with family-inclusive programmes is particularly promising [13, 16, and 19].

## Conclusion

Culturally appropriate antenatal education interventions substantially enhance birth preparedness and complication readiness among pregnant women in LMICs. Programmes that incorporate local languages, context-specific examples, family engagement, and mobile/digital platforms show the greatest effectiveness, improving knowledge, preparedness practices, and institutional delivery rates. Scaling such interventions represents a low-cost, high-impact strategy for advancing maternal health and reducing preventable maternal mortality, aligning with Sustainable Development Goal 3.1. Further robust, long-term evaluations are needed to optimise delivery strategies and ensure equitable access across LMIC populations [1, 16–120].

### Recommendations

Practice: Embed GOPE/mobile tools in routine ANC per WHO 2016/2025, training midwives in cultural adaptation (e.g., local proverbs for danger signs). Prioritise male involvement sessions to boost uptake 20–30% [1, 20].In Nigeria, integrate via NPHCDA platforms for Onitsha/Anambra scale-up.

Policy: LMIC ministries fund context-specific packages; Nigeria’s NHIS reimburses digital ANC; monitors via DHS surveys [27].

Research: Cluster-RCTs evaluating long-term (12-month) perinatal outcomes/costs; equity analyses (rural/indigenous); AI/digital personalisation; West African trials.

### Implications

Overall, this review positions culturally tailored education as a high-impact, low-cost lever for SDG 3.1, potentially averting 10–20% maternal deaths if scaled [1].

## Supplementary Information

Supplementary Material 1.

Supplementary Material 2.

## Data Availability

All data generated or analysed during this study are included in this manuscript and its supplementary information files.
