# Supplementary material for: Effectiveness of culturally appropriate antenatal education packages to improve birth preparedness and complication readiness in low- and middle-income countries: a systematic review
Source: BMC Pregnancy Childbirth. 2026 May 18;26:741. doi: 10.1186/s12884-026-09265-0 (PMC13347876; doi:10.1186/s12884-026-09265-0)
Supplement: Supplementary file 1 — Supplementary Material 1. [file 12884_2026_9265_MOESM1_ESM.docx]

**Supplementary Material 1: Search Terms and Search Strategies**

A comprehensive electronic search was conducted across multiple databases to identify studies evaluating antenatal education interventions aimed at improving Birth Preparedness and Complication Readiness (BPCR) among pregnant women in low- and middle-income countries (LMICs). Searches were limited to studies published between **January 2015 and July 2024,** written in **English,** and involving **human participants.**

**PubMed**

("antenatal education"[Title/Abstract] OR "prenatal education"[Title/Abstract]

OR "antenatal health education"[Title/Abstract] OR "maternal health education"[Title/Abstract])

AND("birth preparedness"[Title/Abstract] OR "complication readiness"[Title/Abstract]

OR "birth preparedness and complication readiness"[Title/Abstract] OR BPCR[Title/Abstract])

AND(pregnant*[Title/Abstract] OR maternal*[Title/Abstract] OR women[Title/Abstract])

AND("low-income countries"[Title/Abstract] OR "middle-income countries"[Title/Abstract]

OR LMIC*[Title/Abstract] OR "developing countries"[Title/Abstract])

**Scopus**

(TITLE-ABS-KEY("antenatal education") OR TITLE-ABS-KEY("prenatal education")

OR TITLE-ABS-KEY("maternal health education"))

AND(TITLE-ABS-KEY("birth preparedness") OR TITLE-ABS-KEY("complication readiness")

OR TITLE-ABS-KEY("birth preparedness and complication readiness") OR TITLE-ABS-KEY(BPCR))

AND(TITLE-ABS-KEY(pregnant*) OR TITLE-ABS-KEY(maternal*) OR TITLE-ABS-KEY(women)) AND(TITLE-ABS-KEY("low-income countries") OR TITLE-ABS

KEY("middle-income countries") OR TITLE-ABS-KEY(LMIC*) OR TITLE-ABS-KEY("developing countries")

**Web of Science**

TS=("antenatal education" OR "prenatal education" OR "maternal health education")

ANDTS=("birth preparedness" OR "complication readiness"

OR "birth preparedness and complication readiness" OR BPCR)

ANDTS=(pregnant* OR maternal* OR women)

ANDTS=("low-income countries" OR "middle-income countries" OR LMIC* OR "developing countries")

**CINAHL**

("antenatal education" OR "prenatal education" OR "maternal health education")

AND("birth preparedness" OR "complication readiness"

OR "birth preparedness and complication readiness" OR BPCR)

AND(pregnant* OR maternal* OR women)

AND("low-income countries" OR "middle-income countries" OR LMIC* OR "developing countries")

**AJOL and Google Scholar**

Simplified keyword combinations were used due to search engine limitations:

Antenatal education AND birth preparedness AND complication readiness AND LMICs

**Supplementary Material 2: Quality Assessment Tool**

The methodological quality of included studies was assessed using the **Joanna Briggs Institute (JBI) Critical Appraisal Checklist,** adapted for cross-sectional, quasi-experimental, cohort, and community-based intervention studies.

**JBI Critical Appraisal Criteria**

1. Was the sample frame appropriate to address the target population?
2. Were study participants sampled appropriately?
3. Was the sample size adequate?
4. Were the study subjects and the setting described in detail?
5. Was the data analysis conducted with sufficient coverage of the identified sample?
6. Were valid methods used for the measurement of outcomes?
7. Were outcomes measured in a standard, reliable way for all participants?
8. Was an appropriate statistical analysis used?
9. Was the response rate adequate, and if not, was it managed appropriately?

Responses were recorded as **Yes, No,** or **Unclear**.

**Risk of Bias Assessment Summary**

| **Study ID** | **Q1** | **Q2** | **Q3** | **Q4** | **Q5** | **Q6** | **Q7** | **Q8** | **Q9** | **Total** |
| --- | --- | --- | --- | --- | --- | --- | --- | --- | --- | --- |
| Masoi & Kibusi, 2019 | Yes | Yes | Yes | Yes | Yes | Yes | Yes | Unclear | Yes | 8 |
| Akinwaare & Oluwatosin, 2023 | Yes | Yes | Yes | Yes | Yes | Yes | Yes | Yes | Yes | 9 |
| Letose et al., 2020 | Yes | Yes | Yes | Yes | No | Yes | Yes | No | Yes | 7 |
| Moinuddin et al., 2017 | Yes | Yes | Yes | Yes | Yes | Yes | Yes | Unclear | Yes | 8 |
| Shimpuku et al., 2019 | Yes | Yes | Yes | Yes | Yes | Yes | No | Yes | Yes | 8 |
| Debelie et al., 2021 | Yes | Yes | Yes | Yes | Yes | Yes | Yes | No | Yes | 8 |
| Girma et al., 2022 | Yes | Yes | Yes | Yes | No | Yes | Yes | No | Yes | 7 |
| Ijang et al., 2021 | Yes | Yes | Yes | Yes | Yes | Yes | No | Yes | Yes | 8 |
| Ihomba et al., 2020 | Yes | Yes | Yes | Yes | Yes | Yes | Yes | No | Yes | 8 |
| Obionu et al., 2022 | Yes | Yes | Yes | Yes | No | Yes | Yes | No | Yes | 7 |
| Imaralu et al., 2020 | Yes | Yes | Yes | Yes | Yes | Yes | Yes | No | Yes | 8 |

**Overall risk of bias:** Low to Moderate across included studies.

**Supplementary Material 3: PRISMA 2020 Checklist**

| **PRISMA 2020 Checklist**  **Section and Topic** | **Item #** | **Checklist item** | **Location where the item is reported** |
| --- | --- | --- | --- |
| **TITLE** |  |  |  |
| Title | 1 | Identify the report as a systematic review | Title page |
| **ABSTRACT** |  |  |  |
| Abstract | 2 | See the PRISMA 2020 for Abstracts checklist | Abstract |
| **INTRODUCTION** |  |  |  |
| Rationale | 3 | Describe the rationale for the review in the context of existing knowledge | Introduction (pp. 3–5) |
| Objectives | 4 | Provide an explicit statement of the objective(s) or question(s) the review addresses | Introduction (pp. 5–6) |
| **METHODS** |  |  |  |
| Eligibility criteria | 5 | Specify inclusion and exclusion criteria and how studies were grouped | Methods – Eligibility Criteria (pp. 6–7) |
| Information sources | 6 | Specify all databases and date last searched | Methods – Information Sources (pp. 7–8) |
| Search strategy | 7 | Present full search strategies including limits | Methods – Search Strategy (pp. 7–8; Appendix I) |
| Selection process | 8 | Describe study screening and selection process | Methods – Study Selection (p. 8) |
| Data collection process | 9 | Describe data extraction process | Methods – Data Extraction (pp. 9–10) |
| Data items | 10a | Define outcomes for which data were sought | Methods – Outcomes (pp. 9–11) |
|  | 10b | Define other variables collected | Methods – Variables (pp. 9–11) |
| Risk of bias assessment | 11 | Describe risk of bias assessment tools | Methods – Risk of Bias Assessment (pp. 10–11) |
| Effect measures | 12 | Specify effect measures used | Methods – Data Synthesis (pp. 10–11) |
| Synthesis methods | 13a–f | Describe synthesis methods and rationale | Methods – Synthesis (pp. 10–11) |
| Reporting bias assessment | 14 | Describe assessment of reporting bias | Not assessed (stated in Methods) |
| Certainty assessment | 15 | Describe certainty assessment | Methods – Certainty of Evidence (pp. 10–11) |
| **RESULTS** |  |  |  |
| Study selection | 16a | Describe search and selection results | Results – Study Selection (pp. 11–12; PRISMA Flow Diagram) |
|  | 16b | Describe excluded studies with reasons | Results – Excluded Studies (pp. 11–12) |
| Study characteristics | 17 | Present characteristics of included studies | Table 1 |
| Risk of bias in studies | 18 | Present risk of bias assessments | Table 1; Results (p. 12) |
| Results of individual studies | 19 | Present outcomes for each study | Results (pp. 12–19) |
| Results of syntheses | 20a–d | Summarise and synthesise findings | Results (pp. 12–19) |
| Reporting biases | 21 | Present assessment of reporting bias | Results (pp. 12–19) |
| Certainty of evidence | 22 | Present certainty of evidence | Results (pp. 12–19) |
| **DISCUSSION** |  |  |  |
| Discussion | 23a | Interpret results in context | Discussion (p. 20) |
|  | 23b | Discuss limitations of evidence | Discussion (p. 23) |
|  | 23c | Discuss limitations of review | Discussion (p. 23) |
|  | 23d | Discuss implications | Discussion (pp. 22–23) |
| **OTHER INFORMATION** |  |  |  |
| Registration and protocol | 24a | State registration or lack thereof | Methods – Protocol (p. 6) |
|  | 24b | Indicate protocol access | Not applicable |
|  | 24c | Describe amendments | Methods – Protocol (p. 6) |
| Support | 25 | Describe funding/support | Declarations (p. 23) |
| Competing interests | 26 | Declare competing interests | Declarations (p. 23) |
| Availability of data | 27 | Describe data availability | Declarations (p. 23) |
