# Supplementary material for: Effectiveness of culturally appropriate antenatal education packages to improve birth preparedness and complication readiness in low- and middle-income countries: a systematic review
Source: BMC Pregnancy Childbirth. 2026 May 18;26:741. doi: 10.1186/s12884-026-09265-0 (PMC13347876; doi:10.1186/s12884-026-09265-0)
Supplement: Supplementary file 2 — Supplementary Material 2. [file 12884_2026_9265_MOESM2_ESM.docx]

**APPENDIX E: PRISMA FLOW DIAGRAM OF STUDY SELECTION**

:

Full-text articles excluded (n = 61):

Not conducted in LMICs (n = 18)

Did not report BPCR outcomes (n = 21)

Inappropriate study design (n = 12)

Not peer-reviewed or full text unavailable (n = 10)

Records screened (n = 964)

Records Identified through database search (n = **1,142)**

Articles after duplicates removal (n = **964)**

Full-text articles assessed for eligibility (n = 72)

Studies included in the systematic review = 11

Meta-analysis (**Not applicable)**

Other sources (google search, grey literature: n = 126)

Articles excluded (n = **892)**

Articles removed before screening (duplicate records: n = **304)**

Identification

Screening

Eligibility

Included

**Fig. 1. PRISMA flow diagram of study selection process**
